# The Second and Third Hemoglobin Kansas Cases in the Turkish Population

**DOI:** 10.4274/tjh.2016.0297

**Published:** 2017-03-01

**Authors:** Zeynep Kayra Tanrıverdi, Arzu Akyay, Aşkın Şen, Çağatay Taşkapan, Ünsal Özgen

**Affiliations:** 1 İnönü University Faculty of Medicine, Department of Pediatrics, Malatya, Turkey; 2 İnönü University Faculty of Medicine, Department of Pediatric Hematology and Oncology, Malatya, Turkey; 3 Fırat University Faculty of Medicine, Department of Medical Genetics, Elazığ, Turkey; 4 İnönü University Faculty of Medicine, Department of Biochemistry, Malatya, Turkey

**Keywords:** Abnormal hemoglobins, Hb Kansas

## TO THE EDITOR,

We read with great interest the article by Keser et al. [1] regarding the first observation of hemoglobin Kansas in Turkey. The authors described a patient from Malatya as the first case of hemoglobin Kansas in the Turkish population. After the publication of this paper, we had two other hemoglobin Kansas cases from the Malatya region.

### Case 1:

**Figure 1:**
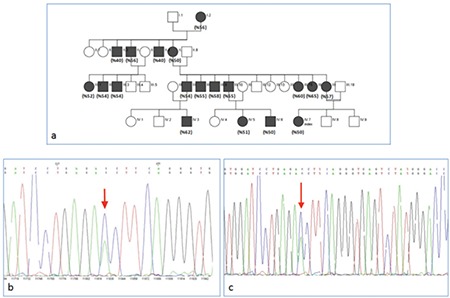
a) Family tree of the patients (transcutaneous oxygen saturations of the affected individuals are shown in parentheses); b) hemoglobin Kansas in DNA sequencing of case 1; c) hemoglobin Kansas in DNA sequencing of case 2.

**Figure 2:**
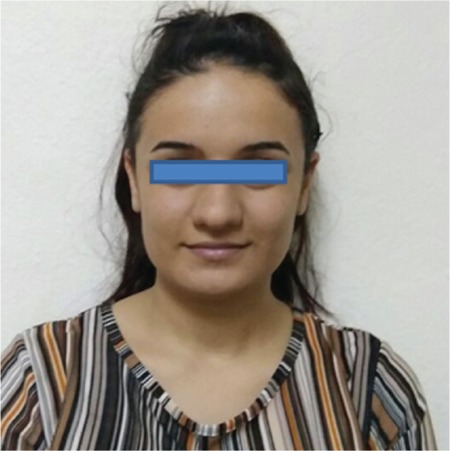
A photograph of case 1 showing cyanosis of her lips.

A 16-year-old female patient was admitted with the complaint of cyanosis of her lips and nails since birth, but she had no problems in her daily life. In her family history, there were other relatives who had the same complaints (Figure 1a). Physical examination of our patient indicated slight cyanosis of her lips, nail beds, and skin (Figure 2). Other system examinations were normal. Transcutaneous oxygen saturation was detected as 50%. Her complete blood count, electrocardiogram, echocardiogram, methemoglobin level, and peripheral blood smear were normal. In blood gas values, pH was 7.39, PCO_2_ was 41.1 mmHg, PO_2_ was 66.3 mmHg, and the P50 value was 66.94 (normal value: 24-29). Hemoglobin electrophoresis revealed HbA1 of 56.3%, HbA2 of 43.5%, and HbF of 2%. In beta-globulin gene DNA sequence analysis, c.308 A>C (β102(G4) Asn>Thr) (Hb Kansas) mutation was detected (Figure 1b).

### Case 2:

A 43-year-old patient, the mother of Case 1, was admitted with the same complaints as her daughter. Transcutaneous oxygen saturation showed low oxygen levels (PO_2_ 57%). Complete blood count, blood chemistry, and cardiac echocardiography were within normal limits. High-performance liquid chromatography results were as follows: HbA1 57.2%, HbA2 42.5%, HbF 0.2%. DNA sequencing revealed the same A to C substitution at nucleotide position 308 as in the first case (Figure 1c).

Hemoglobin Kansas is a rare, unstable, abnormal hemoglobin variant with low oxygen affinity in which asparagine is replaced with threonine in the 102^nd^ position of the β-globin chain [2,3]. In these patients, hemoglobin leaves more than the normal amount of oxygen to extrapulmonary tissues. Therefore, tissues get oxygenated even at low hematocrit levels and patients appear to be healthy. However, cyanosis is seen because the unsaturated hemoglobin amount in the capillaries and veins is higher than 5 g/dL [4]. The P50 values of these patients are also high [5,6].

In total, six hemoglobin Kansas cases were reported from 1968 to date in the world literature [2,3,4,5]. The first hemoglobin Kansas case in Turkey was reported in 2015 [1]. Our patients and 17 other family members who had the same phenotype are more than all of the reported cases in the world literature. We could not perform hemoglobin electrophoresis and genetic evaluations of the other 17 family members because they were living in other cities. However, these patients had low transcutaneous oxygen saturations, as shown in parentheses in Figure 1a. Hemoglobin Kansas and other unstable hemoglobinopathies with low oxygen affinity should be considered in the differential diagnosis of patients with unexplained peripheral cyanosis.
